# Diphtheria Anti-Toxin

**Published:** 1898-06-11

**Authors:** 


### DIPHTHERIA ANTI-TOXIN.

At the last meeting of the Clinical Society the report of the Diphtheria Anti-toxin Committee was presented, and an abstract of it was read by Dr. Church, the chairman of the committee. Dr. Goodall, of the Eastern Hospital of the Metropolitan Asylums Board, had been able to place 475 cases at the disposal of the committee, but with the exception of these the report was founded upon cases occurring in voluntary hospitals. The laryngeal cases are very interesting, confirming what is already known as to the great utility of anti-toxin in lessening the necessity for tracheotomy. Of the cases which presented symptoms more or less severe of laryngeal affection nearly one-half escaped the operation, a much larger proportion than is usually the case in laryngeal diphtheria treated in other ways. Moreover, the mortality after operation amounted only to 36·0 per cent., as opposed to 71·6 per cent. in the control series compiled from the records of the general hospitals before the introduction of anti-toxin, and this lessened mortality largely occurred among the children under five years of age, the period of life in which diphtheria is most fatal. In regard to the suggestion so often made that anti-toxin is itself injurious, the committee say that the closest investigation has failed to discover any connection between the occurrence of paralysis and the amount of anti-toxin injected, nor did the period of the disease at which it was first used appear to exercise any influence on the occurrence of paralytic symptoms. Some rash appeared in nearly a third of the cases. This could not be associated with either the age of the patient, the amount of the anti-toxin, nor the period of the disease at which it was used. In no instance did it appear to have had any bearing on the ultimate result of the case. The percentage of deaths from suppression of urine was practically the same in the anti-toxin and the non-antitoxin series, and as a general result it was stated that in the cases treated with anti-toxin, not only is the mortality notably lessened, but the duration of life in fatal cases is prolonged.

